# Sensitivity of the clinical high-risk and familial high-risk approaches for psychotic disorders – a systematic review and meta-analysis

**DOI:** 10.1017/S0033291724003520

**Published:** 2025-02-12

**Authors:** Animesh Talukder, Ioanna Kougianou, Colm Healy, Ulla Lång, Valentina Kieseppä, Maria Jalbrzikowski, Kirstie O’Hare, Ian Kelleher

**Affiliations:** 1 Centre for Clinical Brain Sciences, Division of Psychiatry, University of Edinburgh, Edinburgh, UK; 2 School of Medicine, University College Dublin, Dublin, Ireland; 3 Faculty of Medicine, University of Oulu, Oulu, Finland; 4 Department of Psychiatry and Behavioral Sciences, Boston Children’s Hospital, Boston, MA, USA; 5 Department of Psychiatry, Harvard Medical School, Boston, MA, USA; 6 St John of God Hospitaller Services Group, Hospitaller House, Dublin, Ireland; 7 Discipline of Psychiatry and Mental Health, University of New South Wales, Sydney, Australia

**Keywords:** clinical high risk, familial risk, psychosis, schizophrenia

## Abstract

**Background:**

Psychosis prediction has been a key focus of psychiatry research for over 20 years. The two dominant approaches to identifying psychosis risk have been the clinical high-risk (CHR) and the familial high-risk (FHR) approaches. To date, the real-world sensitivity of these approaches – that is, the proportion of all future psychotic disorders in the population that they identify – has not been systematically reviewed.

**Methods:**

We systematically reviewed and meta-analysed studies in MEDLINE, Embase, PsychINFO, and Web of Science (from inception until September 2024) that reported data on the sensitivity of CHR and FHR approaches – i.e., individuals with a psychosis diagnosis preceded by a CHR diagnosis or a history of parental psychosis (PROSPERO: CRD42024542268).

**Results:**

We identified four CHR studies and four FHR studies reporting relevant data. The pooled estimate of the sensitivity of the CHR approach was 6.7% (95% CI: 1.5–15.0%) and of the FHR approach was 6.5% (95% CI: 4.4–8.9%). There was a high level of heterogeneity between studies. Most FHR studies had a low risk of bias, but most CHR studies had a high risk of bias.

**Conclusion:**

Pooled data suggest that CHR and FHR approaches, each, capture only about 6–7% of future psychotic disorders. These findings demonstrate the need for additional approaches to identify risk for psychosis.

## Introduction

Psychotic disorders, such as schizophrenia, are characterised by hallucinations, delusions, diminished emotional expression, low motivation, and disorganized speech and behaviour (American Psychiatric Association, 2013; World Health Organization, 1992). They typically have an onset in late adolescence and early adulthood and are frequently chronic with high levels of disability (Díaz-Caneja et al., 2015; Olin & Mednick, 1996). Early detection and intervention for psychotic disorders is known to improve outcomes (Correll et al., 2018).

A major focus of psychiatric research over the past two decades has been to move beyond detection in the early stages of psychosis and to identify people at risk of psychosis before the onset of illness (Fusar-Poli et al., 2020). To date, there have been two dominant approaches to psychosis prediction and prevention research: the clinical high-risk (CHR) approach and the familial high-risk (FHR) approach to psychosis.

The CHR approach – also known as the at-risk mental state (ARMS) or the ultra-high-risk (UHR) approach – usually involves identifying individuals at risk of psychosis based on the presence of one or more of the following criteria: (1) attenuated psychotic symptoms, (2) frank yet brief and intermittent psychotic symptoms, and (3) first-degree-relative of someone with psychosis coupled with a marked functional decline in the past year (Fusar-Poli et al., 2013; Yung & Nelson, 2013).

A systematic review of CHR studies found that 29% of individuals meeting CHR criteria transitioned to psychotic disorders in the following two years (Fusar-Poli et al., 2012), although there is considerable variation in transition rates between studies depending on the specific CHR criteria applied, the length of the follow-up period, and the population from which recruitment occurred (Conrad et al., 2017; Fusar-Poli et al., 2012; Malla et al., 2018; Schultze-Lutter et al., 2015a; Welsh & Tiffin, 2014).

The FHR approach, on the other hand, involves identifying individuals at risk for psychosis based solely on having one or more relatives (especially first-degree relatives) with a history of psychotic disorder (Fusar-Poli et al., 2021). Individuals meeting FHR criteria are at an increased risk of developing psychotic disorders (Agerbo et al., 2015; Rasic, Hajek, Alda, & Uher, 2014; Uher et al., 2023), with a recent systematic review finding an absolute lifetime psychosis risk of 8% among offspring who had parents with a history of psychotic disorder (Uher et al., 2023).

While it is well-established that individuals meeting CHR or FHR criteria have an increased risk of future psychosis, only recently have researchers begun to assess the sensitivity of these approaches for capturing psychosis risk. That is, what proportion of future psychosis diagnoses in the population are captured by the CHR and the FHR approaches. This is important because it informs us about the upper limit of psychosis cases that could be prevented using these approaches if we had an effective preventive intervention (Kelleher, 2023; Lång et al., 2022). We aimed to systematically review and meta-analyse studies that reported data on the proportion of future psychosis cases captured by the CHR or the FHR approach.

## Methods

### Search strategy

We followed the Preferred Reporting Items for Systematic Reviews and Meta Analyses (PRISMA) framework (Page et al., 2021) to structure this review. We ruled out a pre-existing review or review protocol on International Prospective Register of Systematic Reviews (PROSPERO) (Booth et al., 2011). Two authors (AT and IKG) searched for published articles on MEDLINE, Embase, PsychINFO and Web of Science (core collection) (from their inception till September 2024). The search was carried out in the full-text field with variations of following keywords: “psychosis,” “schizophrenia,” “at-risk mental state,” “ultra-high risk,” “clinical high risk,” and “familial high risk.” We also used Medical Subject Headings (MeSH) around “psychosis” and “schizophrenia spectrum disorder” on MEDLINE, Embase, and PsychINFO. The search strategy (Supplement 1) was developed in consultation with subject-matter experts (IK, CH, UL, and KOH) in the research team and a research librarian.

### Eligibility criteria

Peer-reviewed and published studies meeting the following criteria were included: (a) the study population being the general population or, in the case of CHR studies, the population attending CHR services, (b) studies reporting the incidence or prevalence of psychosis diagnoses and the proportion of psychosis diagnoses that were preceded by a CHR diagnosis or a family history of psychosis; (c) CHR status assessed through either the Comprehensive Assessment of at Risk Mental States (CAARMS) (Yung et al., 2005) or the Structured Interview for Psychosis Risk Syndromes (SIPS) (T. J. Miller et al., 2003); (d) FHR status assessed in terms of any history of diagnosed psychotic disorder among one or both parents.

Studies were excluded when they met any of the following criteria: commentaries, letters, conference abstracts, editorials, study proposals/protocols, and case studies.

In terms of the CHR approach, we wished to assess real-world sensitivity. That is, looking at populations with existing CHR services, we wished to identify the total proportion of psychotic disorders identified in CHR clinics in those populations. There were other studies that calculated the sensitivity of the CHR approach within specific, highly selected (i.e., biased) samples (Fusar-Poli et al., 2016; Koutsouleris et al., 2021; Papmeyer et al., 2018; Peralta et al., 2019; Schultze-Lutter, Klosterkötter, & Ruhrmann, 2014; Schultze-Lutter et al., 2015b; Schultze-Lutter, Schimmelmann, & Michel, 2021; Schultze-Lutter et al., 2022; Yung et al., 2008, 2006). As these studies do not tell us about the real-world sensitivity of CHR services, and are not generalisable to the population, they were not included in our meta-analysis.

### Screening and extraction

All search results were exported to and de-duplicated on Covidence (‘Covidence Systematic Review Software’, 2024). AT and IKG independently screened the articles against the eligibility criteria, specifying the reason for any exclusion. Studies not identified by the main search but known to the authors were also included. Any disagreements between AT and IKG were discussed with KOH, IK, UL, or CH to reach consensus. AT and IKG extracted data independently on Covidence. The following data were extracted: (a) the study design, (b) demographic characteristics, (c) psychosis diagnostic criteria based on the International Classification of Diseases (ICD) or the Diagnostic and Statistical Manual of Mental Disorders (DSM) codes, (d) instruments used to ascertain CHR and FHR statuses, and (e) data concerning the sensitivity of CHR and FHR approaches.

### Risk of bias assessment

AT and IKG independently appraised the included studies for the risk of bias using a modified version of the Newcastle-Ottawa Quality Assessment Form for Cohort Studies (Wells et al., 2021). The following aspects were assessed in relation to the ‘selection’ and the ‘outcome’ domains of the tool: (a) representativeness of the exposed cohort (i.e., subjects with CHR/FHR), (b) selection of the non-exposed cohort (i.e., subjects with no CHR/FHR), (c) ascertainment of exposure (i.e., CHR/FHR status) and outcome (i.e., psychosis status among index subjects), (d) adequacy of follow-up time (i.e., for how long the subjects were followed up for the psychosis outcome), and (e) follow-up response rate.

The possible scores range from 0 to 7. We graded the studies in following categories based on their domain-specific score (Wells et al., 2021): (a) ‘low risk’ for a score of 3 or 4 in selection domain AND 2 or 3 in outcome domain, (b) ‘moderate risk’ for a score of 2 in selection domain AND 2 or 3 stars in outcome domain, and (c) ‘high risk’ for a score or 0 or 1 in selection domain OR 0 or 1 in outcome domain (Supplement 2).

### Statistical analysis

#### Estimating the sensitivity of CHR and FHR

We meta-analysed the sensitivity proportions to present pooled sensitivity point estimates, along with 95% confidence intervals [CI]) calculated using Wilson’s Score method (Newcombe, 1998), for CHR and FHR separately using Stata/SE 18 (‘*meta’* package). We employed a random-effects model assuming that different studies estimated different (yet related) sensitivity estimands, since the assumption of one true estimand may not hold for prevalence or proportion data (Munn, Moola, Lisy, Riitano, & Tufanaru, 2015).

The raw proportions were transformed using the Freeman-Tukey double-arcsine transformation approach to improve their statistical properties (Barendregt, Doi, Lee, Norman, & Vos, 2013; Freeman & Tukey, 1950). To weigh each study, we used the inversed variance of each transformed proportion of that respective study (Borenstein, Hedges, Higgins, & Rothstein, 2010), following the Sidik-Jonkman approach (Deeks, Higgins, & Altman, 2019; Sidik & Jonkman, 2002). The pooled estimates were then back-transformed to proportions (J. J. Miller, 1978) and presented with forest plots.

#### Assessing heterogeneity

We investigated the evidence of heterogeneity in the pooled estimates across studies, i.e., − whether the variation across studies exceeds that expected from random error alone – by computing Cochran’s *χ^2^* test statistic (Cochran, 1954) and the corresponding *p*-value. We considered a *p*-value of <0.10 as statistically significant evidence of heterogeneity (Deeks et al., 2019).

We quantified statistical heterogeneity through the *I^2^* statistic; i.e., the proportion of the variability that is attributable to heterogeneity rather than to random error (Higgins & Thompson, 2002). We also presented the *τ^2^* statistic which represents the variance of the distribution of the underlying estimands across studies (Borenstein et al., 2010), and the *H^2^* statistic, which represents the ratio of the observed variance to the expected variance from random error alone (Higgins & Thompson, 2002).

#### Analyses of sub-groups

When a study was deemed markedly heterogenous in terms of pre-specified characteristics, e.g., CHR/FHR assessment criteria or the risk of bias, we excluded it from the overall meta-analysis and performed a sub-group meta-analysis. The exclusion was considered influential if the sub-group and the overall estimates had non-overlapping CIs (Deeks et al., 2019).

### Registration of the protocol

The protocol was registered on PROSPERO (Booth et al., 2011) on 10th May 2024 (registration number: CRD42024542268).

## Findings

### Selecting eligible studies

The electronic database search retrieved 9,130 unique articles. We also added three studies (Blomström et al., 2016; Debost et al., 2019; Mortensen, Pedersen, & Pedersen, 2010) following expert consultation within the research team. We excluded 9,103 articles after title and abstract screening and 23 after full-text screening. During the full-text screening, the study by Ajnakina et al. (2017) (Ajnakina et al., 2017) was excluded, since it involved a sub-set of one of the samples studied by Fusar-Poli et al. (2017) (Fusar-Poli et al., 2017). Also, since Burke et al. (2022) reported data relating to both CHR and FHR (Burke et al., 2022), we excluded the FHR sample since it involved a help-seeking population referred to a CHR service as opposed to a general population with data on familial risk. One study that experts had identified as possibly relevant was not included as it was ultimately not possible to calculate FHR sensitivity from the available data. This resulted in five eligible studies from our database search (Burke et al., 2022; Fusar-Poli et al., 2017; Healy et al., 2024; Sullivan et al., 2020; Veijola et al., 2013) and two eligible studies from expert consultation (Blomström et al., 2016; Debost et al., 2019). Therefore, in total, we included seven studies (Blomström et al., 2016; Burke et al., 2022; Debost et al., 2019; Fusar-Poli et al., 2017; Healy et al., 2024; Sullivan et al., 2020; Veijola et al., 2013) in the review.

Of the seven included studies, three (Burke et al., 2022; Fusar-Poli et al., 2017; Sullivan et al., 2020) reported data on the sensitivity of the CHR approach, involving four unique samples. Fusar-Poli et al. (2017) reported sensitivity data from two mutually exclusive populations in South London: one from the Lambeth and Southwark boroughs (denoted in this review as Fusar-Poli et al., 2017 *(a)*) and the other from the Croydon and Lewisham boroughs (denoted in this review as Fusar-Poli et al., 2017 *(b)*).

**Figure 1.**
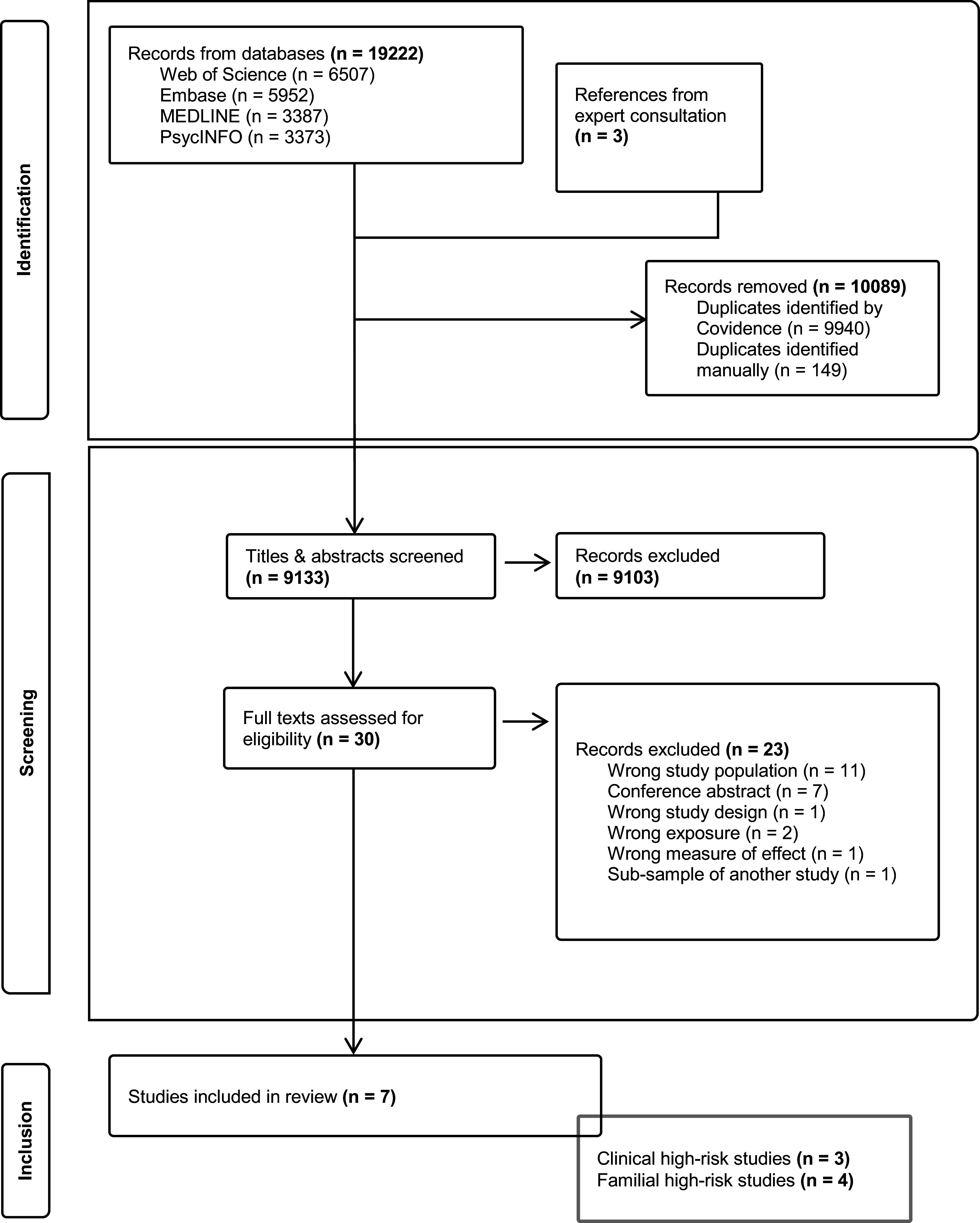
PRISMA flow diagram of the study selection process.

We identified one study that reported on the sensitivity of the FHR approach (Healy et al., 2024). We also identified three additional studies, however, from which it was possible to extract data on FHR sensitivity (Blomström et al., 2016; Debost et al., 2019; Veijola et al., 2013) (Figure 1).

### Description of included studies

#### Baseline characteristics

Six out of the seven included studies were conducted in Northern Europe (United Kingdom (Fusar-Poli et al., 2017; Sullivan et al., 2020), Sweden (Blomström et al., 2016), Finland (Healy et al., 2024; Veijola et al., 2013) and Denmark (Debost et al., 2019)) and one in Australia (Burke et al., 2022). While three of the four FHR studies were based on total population-wide registries (Blomström et al., 2016; Debost et al., 2019; Healy et al., 2024), one of the three CHR studies was based on primary data from a total population-wide cohort (Sullivan et al., 2020), whereas the other two were based on help-seeking populations (Burke et al., 2022; Fusar-Poli et al., 2017).

#### Characteristics of CHR studies

The CHR status was assessed with the CAARMS instrument in two studies (Burke et al., 2022; Fusar-Poli et al., 2012). Sullivan et al. (2020), on the other hand, assessed psychosis-like symptoms through a semi-structured questionnaire, referred as Psychosis-Like-Symptoms Interview (PLIKS); the assessment was then matched to the SIPS criteria, to determine the CHR status at age 18. Regarding psychosis diagnostic criteria, Fusar-Poli et al. (2017) reported ICD-10 codes to ascertain all psychosis diagnoses, whereas Burke et al. (2022) used the DSM-IV criteria to determine all psychosis diagnoses. Sullivan et al. (2020) compared the PLIKS assessment with SIPS-psychosis and CAARMS-psychosis criteria to determine the presence of psychosis. The age of individuals when CHR was determined was 18 years, on average, in two of three CHR studies (Burke et al., 2022; Sullivan et al., 2020), while the other study did not report it (Fusar-Poli et al., 2017) (Table 1).

**Table 1.** Characteristics of clinical high-risk studies

	Fusar-Poli et al. (2017)	Sullivan et al. (2020)	Burke et al. (2022)
Study setting	Help-seeking population visiting SLaM clinic, London, UK	General population in Avon, UK (birth cohort 1991–92)	Help-seeking population visiting Orygen clinic, Melbourne, Australia
Total sample (n)	(a) 33,830, (b) 54,716*	2,804	1,123
**CHR assessment**			
Period when assessed	2008–2015	2009–2010	2012–2016
Age (years)	Not reported	At 18	Mean (SD): 18 ± 2.8
Assessment tool	CAARMS	PLIKS (matched to SIPS criteria)	CAARMS
**Psychosis diagnosis**			
Period when assessed	(a) 1589 days (b) 1588 days (on average, after CHR assessment)*	2009–2015	2–3 years after CHR assessment**
Age (years)	Not reported	18–24	Mean (SD): 19.4 ± 2.8
Assessment tool	CAARMS	PLIKS (matched to SIPS and CAARMS criteria)	CAARMS
Nature of diagnosis	Non-affective psychosis, psychotic disorders due to psychoactive substance use, affective psychosis, bipolar affective disorder with psychotic symptoms, depression with psychotic symptoms	Psychotic disorder defined as a regular occurance of psychotic experience, reported as very distressing or with a very negative impact on social or occupational functioning or leading to professional help-seeking	Schizophrenia, schizophreniform disorder, drug-induced psychosis, psychosis not otherwise specified, not differentiated, brief psychotic disorder, delusional disorder, schizoaffective and bipolar affective disorder, depression with psychosis
Diagnostic classification system	ICD–10 (F20.x (except F20.4/F20.5), F22.x, F23.x, F24, F25.x, F28/F29,F10-F19.5, F30.2, F31.2, F31.5, F32.3/F33.3)	Not applicable	DSM-IV criteria

*Note:* SLaM, South London and Maudsley; CAARMS, Comprehensive Assessment of At-Risk Mental State; PLIKS, Psychosis-Like Symptoms Interview; SIPS, Structured Interview for Psychosis-risk Syndromes; ICD, International Classification of Diseases; DSM, Diagnostic and Statistical Manual of Mental Disorders.

*(a) as Fusar-Poli et al., 2017 (a) and (b) as Fusar-Poli et al., 2017 (b), ** In Orygen, those who enter the CHR clinics are followed up for two years after initial assessment with CAARMS. However, individuals entering CHR clinics at age 15 are eligible to receive care up to age 18 (Burke et al., 2022).

#### Characteristics of FHR studies

We identified one study that reported on the sensitivity of the FHR approach (Healy et al., 2024). We also identified three additional studies from which it was possible to extract data on FHR sensitivity (Blomström et al., 2016; Debost et al., 2019; Veijola et al., 2013). All four studies (Blomström et al., 2016; Debost et al., 2019; Healy et al., 2024; Veijola et al., 2013) defined FHR as any individual with a parental history of a psychotic disorder. However, the studies used different age intervals to assess and assign the FHR status among the offspring. Healy et al. (2024) determined FHR in the offspring at different age cut-offs: at birth, at 5th birthday, at 13th birthday, at 18th birthday, and at any time between their birth and the end of the follow-up period (25–29 years of age). Debost et al. (2019) determined FHR from birth till 15 years of age . Blomström et al. (2016) ascertained FHR between 13 and 33 years of age. Veijola et al. (2013) ascertained FHR from birth till 20 years of age. Three of the four FHR studies reported non-affective psychosis diagnoses as the outcome (Blomström et al., 2016; Debost et al., 2019; Healy et al., 2024) (Table 2).

**Table 2.** Characterisitcs of familial high-risk studies

	Blomström et al. (2016)	Veijola et al. (2013)	Healy et al. (2024)	Debost et al. (2019)
Study setting	Swedish nationwide birth cohort (1978–1997)	Finnish birth cohort from Oulu and Lapland provinces (1985–1986)	Finnish nationwide birth cohort (1987–1992)	Danish nationwide birth cohort (1981–1998)
Total sample (n)	1,971,623	295	368,937	882,813
**FHR assessment**				
Period when assessed	1991–2011	1985–2005	1987–2016	1981–2013
Age of offspring (years)	13–33	0–20	0–30	0–15
FHR defined as	History of parental psychotic diagnoses	History of parental psychotic diagnoses	History of parental psychotic diagnoses	History of parental psychotic diagnoses
Nature of parental psychosis diagnosis	Non-affective psychotic diagnoses extracted from inpatient/outpatient records	Any psychotic diagnoses extracted from inpatient records	Non-affective psychotic diagnoses extracted from inpatient records	Non-affective psychotic diagnoses extracted from inpatient/outpatient or emergency-visit records
Parental psychosis diagnostic codes	ICD–10 (F20-F29)	ICD–8, ICD–9 (295–299), ICD–10 (F20-F33 – excluding non-psychotic mood disorders)	ICD–8 (295,297,298.10–299.99),ICD–9 (297–298),ICD–10 (F20-F29)	ΙCD–8 (295,297, 298.39, 301.83), ICD–10 (F20-F29)
**Psychosis diagnosis among offspring**				
Period when assessed	1991–2011	2007–2010	1987–2016	1996–2013
Age (years)	13–33	20.7–25.3	25–30	15–33
Nature of diagnosis	Non-affective psychotic diagnoses extracted from inpatient/outpatient records	Any psychotic diagnoses using SIPS-psychosis criteria	Non-affective psychotic diagnoses extracted from inpatient/outpatient records	Schizophrenia diagnoses extracted from inpatient/outpatient records
Diagnostic codes	ICD–9 (295, 297, and 298 except 298A and B), ICD–10 (F20–29)	Not applicable (since diagnoses made using SIPS-psychosis criteria)	ICD–10 (F20–29)	ICD–10 (F20.x.)

*Note:* ICD, International classification of diseases; SIPS, Structured Interview for Psychosis-risk Syndromes; FHR, familial high-risk

### Risk of bias in included studies

Two of the three CHR studies (Burke et al., 2022; Fusar-Poli et al., 2017), were found to be at high risk of bias, because their participants were not representative of the average CHR individuals in the community, they may not have performed an independent blind assessment of the outcome (i.e., psychosis), and they did not report the retention rate or whether the retention was adequate at the end of the follow-up. The third CHR study (Sullivan et al., 2020), on the other hand, had a low risk of bias (Supplement 3).

Three of the four FHR studies were found to have a low risk of bias (Blomström et al., 2016; Debost et al., 2019; Healy et al., 2024). The fourth study (Veijola et al., 2013) had a moderate risk of bias since their retention proportion was less than 50%, implying a risk that the participants were non-representative of typical FHR cases in the community. In addition, it was not possible to rule out the absence of a psychosis diagnosis in participants at the start of the follow-up (Veijola et al., 2013) (Supplement 3).

### Meta-analyses

#### Sensitivity of the CHR approach

**Figure 2.**
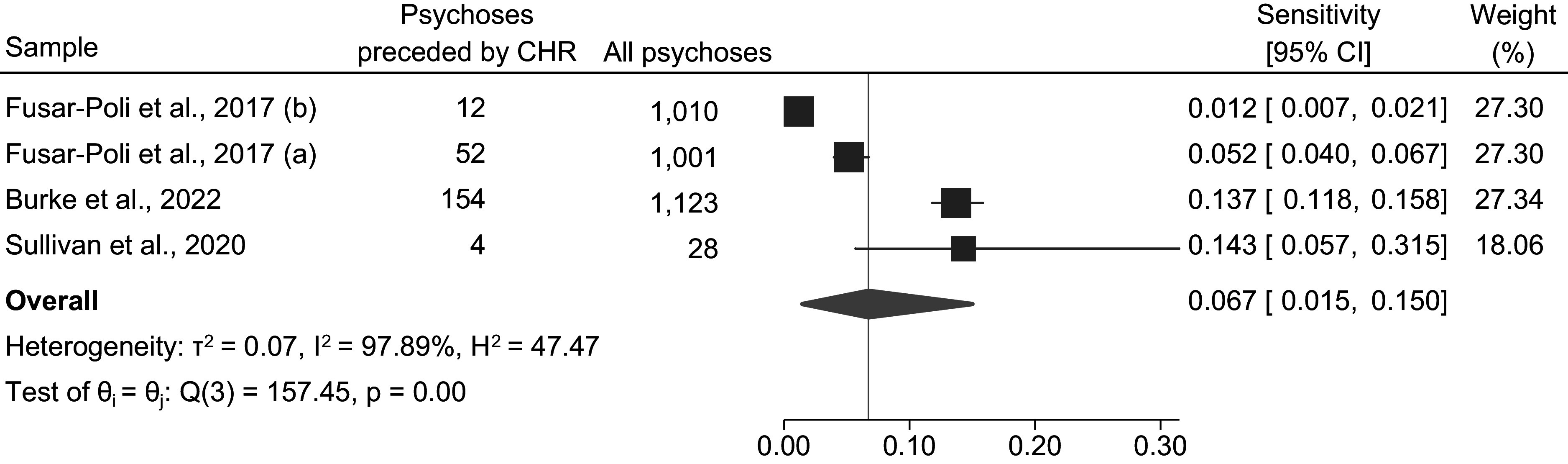
Pooled estimate of sensitivity of the clinical high-risk approach. *Note:* Random Effects Sidik–Jonkman Model; θ: true sensitivity parameter; CHR = Clinical high-risk.

We pooled four sensitivity estimates from CHR studies (Burke et al., 2022; Fusar-Poli et al., 2020; Sullivan et al., 2020). The pooled estimate of the sensitivity of the CHR approach is 0.067 (95% CI: 0.015–0.150), with strong evidence for statistical heterogeneity (*χ^2^*[3] = 157.45, *p* < .001*; I^2^* = 97.89%, *τ^2^* = 0.07, *H^2^* = 47.47) (Figure 2).

#### Sensitivity of the FHR approach

Blomström et al. (2016) reported two sensitivity estimates: one for 288 individuals with a paternal history of psychosis (sensitivity estimate: 0.035) and the other for 420 individuals with a maternal history of psychosis (sensitivity estimate: 0.050). We assumed that there was little overlap between individuals with a history of paternal and maternal psychotic diagnosis, as previously shown by Healy et al (2024), so we combined the two estimates to derive one single estimate (0.085) from that study (Blomström et al., 2016). We considered the lifetime FHR sensitivity estimate from Healy et al. (2024), who reported multiple estimates based on multiple time points for FHR ascertainment.

**Figure 3.**
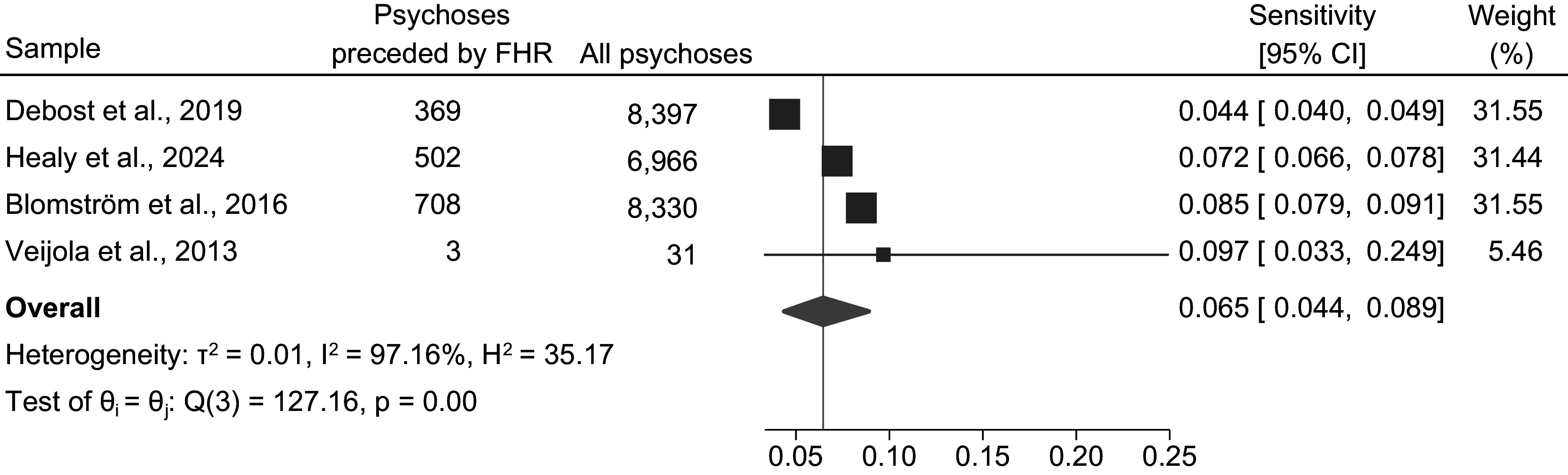
Pooled estimate of sensitivity of the familial high-risk approach *Note:* Random Effects Sidik–Jonkman Model; θ: true sensitivity parameter; FHR = Familial high-risk.

Therefore, we pooled four sensitivity estimates from all four FHR studies (Blomström et al., 2016; Debost et al., 2019; Healy et al., 2024; Veijola et al., 2013). The pooled estimate of the sensitivity of the FHR approach is 0.065 (95% CI: 0.044–0.089), with strong evidence for statistical heterogeneity (*χ^2^*[3] = 127.16, *p* < .001*; I^2^* = 97.16%, *τ^2^* = 0.01, *H^2^* = 35.17) *(Figure 3).*

### Sub-group analysis

#### Sensitivity of the CHR approach based on studies involving CHR services

We also carried out a meta-analysis of studies on “real-world” CHR clinics; that is, studies involving actual CHR services (Burke et al., 2022; Fusar-Poli et al., 2017) (as opposed to the study that actively recruited participants from the general population and applied CHR criteria (Sullivan et al., 2020)). The pooled estimate of the sensitivity of the CHR approach based on those studies is 0.056 (95% CI: 0.007–0.146), with strong evidence for statistical heterogeneity (*χ^2^*[2] = 154.61, *p* < .001*; I^2^* = 98.67%, *τ^2^* = 0.07, *H^2^* = 75.06) (Supplement 4).

#### Sensitivity of the FHR approach based on studies with a low risk of bias

We meta-analysed the FHR studies with a low risk of bias (Blomström et al., 2016; Debost et al., 2019; Healy et al., 2024). The pooled estimate from this sub-group analysis is 0.066 (95% CI: 0.044–0.092), with strong evidence for statistical heterogeneity (*χ^2^*[2] = 126.40, *p* < .001*; I^2^* = 98.32%, *τ^2^* = 0.01, *H^2^* = 59.44) (Supplement 5).

## Discussion

We carried out a systematic review and meta-analysis of studies providing data on the sensitivity of CHR and FHR approaches; that is, of all future psychotic disorders in the population, what proportion do these approaches identify. We identified four CHR samples and four FHR samples reporting relevant data. The pooled point estimate for the sensitivity of the CHR approach was 6.7%. The pooled point estimate for the sensitivity of the FHR approach was 6.5%.

In terms of the CHR paradigm, three of the four included samples involved “real world” CHR services (Burke et al., 2022; Fusar-Poli et al., 2017). The pooled estimate of the sensitivity of the CHR approach from those three samples was 5.6%. The fourth CHR sample (Sullivan et al., 2020) applied CHR criteria to a general population sample (i.e., not in the context of a CHR clinic). In that study, they assessed the general population sample for psychotic symptoms at age 18 and followed them until age 24. This approach still missed a large majority (approx. 86%) of future psychotic disorder diagnoses, demonstrating the limitations of symptom-based approaches even when applied at scale.

In the case of the FHR approach, three of the four studies included total population data and, therefore, likely reflect the true sensitivity of the FHR approach in the population. As with the CHR approach, the FHR approach captured only a small minority of future psychotic disorders. Recent FHR research has also investigated parental mental health service use more broadly (not limited to parental psychotic disorders) to see if this might capture a larger proportion of future psychosis cases in offspring. Specifically, Healy et al. (2024) found that, while 7.2% of all psychotic disorders occurred in the offspring of parents with a history of psychosis, 28.7% of all psychotic disorders occurred in the offspring of parents who had a history of inpatient psychiatric admission (for any reason, not limited to psychosis) (Healy et al., 2024). This highlights opportunities to expand risk detection beyond existing approaches.

Additional approaches to identifying risk for psychosis have included following young people who have presented to the emergency department with self-harm (Bolhuis et al., 2024, 2021) and who have attended child and adolescent mental health services (Lång et al., 2022). In particular, longitudinal research in Finland (Lång et al., 2022) showed that up to half of all psychotic disorder diagnoses emerged in individuals who had, at some stage in childhood (age < 18), attended child and adolescent psychiatry services. Given international variation in the architecture and functioning of child mental health services (Signorini et al., 2017), this finding requires replication outside of Finland but suggests that child psychiatry services represent a promising avenue for future psychosis risk research.

FHR studies varied in the age of the offspring at which FHR status was determined. The study with the lowest sensitivity estimate (4.4%) had determined the FHR status up to age 15 years (Debost et al., 2019), compared to Veijola et al. (2013) up to age 20 years (sensitivity estimate: 9.7%), Healy et al. (2024) up to age 30 years (sensitivity estimate: 7.2%), and Blomström et al. (2016) between 13 and 33 years (sensitivity estimate: 8.5%). Healy et al. (2024) has found that the sensitivity of the FHR approach increases as the age of the offspring at which FHR status is determined increases, highlighting the dynamic nature of this approach. It is, however, important to point out that this study was the only population-based study that specifically aimed to calculate FHR sensitivity. The other population-based FHR studies in our review just reported incidental data that made it possible for us to also calculate FHR sensitivity but without the same fine-grained detail on age cut-offs provided by Healy et al. (2024). There was also variation in terms of the risk of bias; however, a sub-group analysis excluding the one FHR study with a high risk of bias (Veijola et al., 2013) produced a similar estimate (6%) to the main analysis (Supplement 4).

Three of the four CHR samples reported data from “real world” CHR clinics (Burke et al., 2022; Fusar-Poli et al., 2017) but the sensitivity estimates varied across the study settings: 5.2% in Lambeth and Southwark boroughs of South London (Fusar-Poli et al., 2017), 1.2% in Lewisham and Croydon boroughs of South London (Fusar-Poli et al., 2017), and 13.7% in Melbourne (Burke et al., 2022). The difference in these estimates may reflect differences in the catchment population of the clinics, outreach activity, referral systems and waiting times for CHR assessment, and the amount of immigration to and emigration from the catchment areas. For instance, London has a very dynamic migration pattern, with the South London boroughs experiencing a net positive external migration according to the 2021 census (LandTech, 2024). Such a dynamic migration pattern could affect access to services for psychosis due to the lack of a stable healthcare registration, as well as issues specific to immigrant populations, such as cultural stigma, lack of awareness, or language barriers (Pollard & Howard, 2021) – all of which could affect the sensitivity of CHR clinics in identifying individuals at risk of psychosis in these areas.

Disparities in access to mental health services mean that groups such as migrants, minoritised ethnic groups, and people living in socially deprived areas may also be less likely to come into contact with CHR clinics (Ajnakina, David, & Murray, 2019; Ajnakina et al., 2017; Morgan et al., 2006; Steele, Dewa, & Lee, 2007). We found, however, that the sensitivity estimate derived from the study by Sullivan et al. (2020) (14.3%), which screened a general population sample with CHR criteria (Sullivan et al., 2020), was in line with the estimate from the help-seeking sample from the Melbourne PACE clinic (13.7%) (Burke et al., 2022). This suggests that even if there were no barriers to accessing CHR services, the approach would still not capture a large majority of future psychosis cases.

### Strengths and limitations

This review includes studies based on both help-seeking populations and general population-wide registries captured in four major bibliographic databases since their inception. All studies retrieved were conducted either in Northern European countries or Australia, which may limit the generalisability of our results. Further, we did not formally search for grey literature, which may have led to the exclusion of unpublished articles. One CHR study (Fusar-Poli et al., 2017) did not follow up individuals who were rated as CHR negative. This means that the sensitivity estimate for this study should be considered optimistic as it assumes that there were no false negatives (i.e., individuals who went on to develop psychosis) in the CHR negative group. Based on studies that have followed CHR negative individuals over time, this is, however, unlikely (Conrad et al., 2017). The higher the number of false negatives, the lower the true sensitivity would be for that study. We did not include approaches to assessing symptomatic risk other than those using CAARMS or SIPS criteria, such as the basic symptom (BS) approach, as other criteria are not widely used internationally in CHR services (Andreou, Bailey, & Borgwardt, 2019; Thompson, Marwaha, & Broome, 2016).

One FHR study (Blomström et al., 2016) did not report data on the overlap between offspring with maternal and paternal histories of psychosis, meaning that our combined sensitivity estimate for that study may have been overestimated, as anyone with two parents with psychosis would be counted twice in the numerator. However, Healy et al. (2024) found that only 0.3% of individuals with a psychosis diagnosis had both maternal and paternal histories of psychosis (Healy et al., 2024), meaning, it is unlikely that the true sensitivity was substantially overestimated for that individual study (Blomström et al., 2016).

## Conclusions

CHR and FHR approaches have created an important clinical and research focus on psychosis prediction and prevention. The findings of this review show, however, that these strategies identify only a small minority of all individuals who will go on to develop psychotic disorders in the population – just 6–7%, each. These findings highlight the need for additional approaches to psychosis risk detection if we wish to increase the capacity for psychosis prediction and, ultimately, prevention, rather than relying on any single approach.

## Supporting information

Talukder et al. supplementary material Talukder et al. supplementary material

## Supplementary material

The supplementary material for this article can be found at http://doi.org/10.1017/S0033291724003520.

## References

[r1] Agerbo, E., Sullivan, P. F., Vilhjálmsson, B. J., Pedersen, C. B., Mors, O., Børglum, A. D., … Mortensen, P. B. (2015). Polygenic risk score, parental socioeconomic status, family history of psychiatric disorders, and the risk for schizophrenia. JAMA Psychiatry, 72(7), 635. doi: 10.1001/jamapsychiatry.2015.0346 25830477

[r2] Ajnakina, O., David, A. S., & Murray, R. M. (2019). ‘At risk mental state’ clinics for psychosis – an idea whose time has come – and gone! Psychological Medicine, 49(4), 529–534. doi: 10.1017/S0033291718003859 30585562

[r3] Ajnakina, O., Morgan, C., Gayer-Anderson, C., Oduola, S., Bourque, F., Bramley, S., … David, A. S. (2017). Only a small proportion of patients with first episode psychosis come via prodromal services: A retrospective survey of a large UK mental health programme. BMC Psychiatry, 17(1), 308. doi: 10.1186/s12888-017-1468-y 28841826 PMC5574213

[r4] American Psychiatric Association. (2013). Diagnostic and statistical manual of mental disorders (5th ed.). Arlington: American Psychiatric Association.

[r5] Andreou, C., Bailey, B., & Borgwardt, S. (2019). Assessment and treatment of individuals at high risk for psychosis. BJPsych Advances, 25(3), 177–184. doi: 10.1192/bja.2019.3

[r6] Barendregt, J. J., Doi, S. A., Lee, Y. Y., Norman, R. E., & Vos, T. (2013). Meta-analysis of prevalence. Journal of Epidemiology and Community Health, 67(11), 974–978. doi: 10.1136/jech-2013-203104 23963506

[r7] Blomström, Å., Karlsson, H., Gardner, R., Jörgensen, L., Magnusson, C., & Dalman, C. (2016). Associations between maternal infection during pregnancy, childhood infections and the risk of subsequent psychotic disorder—a Swedish cohort study of nearly 2 million individuals. Schizophrenia Bulletin. doi: 10.1093/schbul/sbv112 PMC4681563 26303935

[r8] Bolhuis, K., Ghirardi, L., Kuja-Halkola, R., Lång, U., Cederlöf, M., Metsala, J., … Kelleher, I. (2024). Risk of psychosis among individuals who have presented to hospital with self-harm: A prospective nationwide register study in Sweden. Schizophrenia Bulletin, 50(4), 881–890. doi: 10.1093/schbul/sbae002 38243843 PMC11283185

[r9] Bolhuis, K., Lång, U., Gyllenberg, D., Kääriälä, A., Veijola, J., Gissler, M., & Kelleher, I. (2021). Hospital presentation for self-harm in youth as a risk marker for later psychotic and bipolar disorders: A cohort study of 59 476 finns. Schizophrenia Bulletin, 47(6), 1685–1694. doi: 10.1093/schbul/sbab061 33991091 PMC8530384

[r10] Booth, A., Clarke, M., Ghersi, D., Moher, D., Petticrew, M., & Stewart, L. (2011). An international registry of systematic-review protocols. The Lancet, 377(9760), 108–109. doi: 10.1016/S0140-6736(10)60903-8 20630580

[r11] Borenstein, M., Hedges, L. V., Higgins, J. P. T., & Rothstein, H. R. (2010). A basic introduction to fixed-effect and random-effects models for meta-analysis. Research Synthesis Methods, 1(2), 97–111. doi: 10.1002/jrsm.12 26061376

[r12] Burke, T., Thompson, A., Mifsud, N., Yung, A. R., Nelson, B., McGorry, P., & O’Donoghue, B. (2022). Proportion and characteristics of young people in a first-episode psychosis clinic who first attended an at-risk mental state service or other specialist youth mental health service. Schizophrenia Research, 241, 94–101. doi: 10.1016/j.schres.2021.12.035 35101839

[r13] Cochran, W. G. (1954). The combination of estimates from different experiments. Biometrics, 10(1), 101. doi: 10.2307/3001666

[r14] Conrad, A. M., Lewin, T. J., Sly, K. A., Schall, U., Halpin, S. A., Hunter, M., & Carr, V. J. (2017). Utility of risk-status for predicting psychosis and related outcomes: Evaluation of a 10-year cohort of presenters to a specialised early psychosis community mental health service. Psychiatry Research, 247, 336–344. doi: 10.1016/j.psychres.2016.12.005 27984822

[r15] Correll, C. U., Galling, B., Pawar, A., Krivko, A., Bonetto, C., Ruggeri, M., … Kane, J. M. (2018). Comparison of early intervention services vs treatment as usual for early-phase psychosis. JAMA Psychiatry, 75(6), 555. doi: 10.1001/jamapsychiatry.2018.0623 29800949 PMC6137532

[r16] Covidence systematic review software. (2024). Retrieved 23 April 2024, from https://www.covidence.org/

[r17] Debost, J.-C., Larsen, J. T., Munk-Olsen, T., Mortensen, P. B., Agerbo, E., & Petersen, L. V. (2019). Childhood infections and schizophrenia: The impact of parental SES and mental illness, and childhood adversities. Brain, Behavior, and Immunity, 81, 341–347. doi: 10.1016/j.bbi.2019.06.031 31247291

[r18] Deeks, J. J., Higgins, J. P., & Altman, D. G. (2019). Analysing data and undertaking meta-analyses. In Cochrane handbook for systematic reviews of interventions (pp. 241–284). Wiley. doi: 10.1002/9781119536604.ch10

[r19] Díaz-Caneja, C. M., Pina-Camacho, L., Rodríguez-Quiroga, A., Fraguas, D., Parellada, M., & Arango, C. (2015). Predictors of outcome in early-onset psychosis: A systematic review. NPJ Schizophrenia, 1(1), 14005. doi: 10.1038/npjschz.2014.5 27336027 PMC4849440

[r20] Freeman, M. F., & Tukey, J. W. (1950). Transformations related to the angular and the square root. The Annals of Mathematical Statistics, 21(4), 607–611. doi: 10.1214/aoms/1177729756

[r21] Fusar-Poli, P., Bonoldi, I., Yung, A. R., Borgwardt, S., Kempton, M. J., Valmaggia, L., … McGuire, P. (2012). Predicting psychosis: Meta-analysis of transition outcomes in individuals at high clinical risk. Archives of General Psychiatry, 69(3), 220–229. doi: 10.1001/archgenpsychiatry.2011.1472 22393215

[r22] Fusar-Poli, P., Borgwardt, S., Bechdolf, A., Addington, J., Riecher-Rössler, A., Schultze-Lutter, F., … Yung, A. (2013). The psychosis high-risk state. JAMA Psychiatry, 70(1), 107. doi: 10.1001/jamapsychiatry.2013.269 23165428 PMC4356506

[r23] Fusar-Poli, P., Cappucciati, M., Borgwardt, S., Woods, S. W., Addington, J., Nelson, B., … McGuire, P. K. (2016). Heterogeneity of psychosis risk within individuals at clinical high risk. JAMA Psychiatry, 73(2), 113. doi: 10.1001/jamapsychiatry.2015.2324 26719911

[r24] Fusar-Poli, P., Correll, C. U., Arango, C., Berk, M., Patel, V., & Ioannidis, J. P. A. (2021). Preventive psychiatry: A blueprint for improving the mental health of young people. World Psychiatry, 20(2), 200–221. doi: 10.1002/wps.20869 34002494 PMC8129854

[r25] Fusar-Poli, P., Rutigliano, G., Stahl, D., Davies, C., Bonoldi, I., Reilly, T., & McGuire, P. (2017). Development and validation of a clinically based risk calculator for the transdiagnostic prediction of psychosis. JAMA Psychiatry, 74(5), 493. doi: 10.1001/jamapsychiatry.2017.0284 28355424 PMC5470394

[r26] Fusar-Poli, P., Salazar de Pablo, G., Correll, C. U., Meyer-Lindenberg, A., Millan, M. J., Borgwardt, S., … Arango, C. (2020). Prevention of psychosis: Advances in detection, prognosis, and intervention. JAMA Psychiatry, 77(7), 755. doi: 10.1001/jamapsychiatry.2019.4779 32159746

[r27] Healy, C., Lång, U., O’Hare, K., Veijola, J., O’Connor, K., Lahti-Pulkkinen, M., … Kelleher, I. (2024). Sensitivity of the familial high-risk approach for the prediction of future psychosis: A total population study. World Psychiatry, 23(3), 432–7. doi: 10.1002/wps.21243 39279372 PMC11403180

[r28] Higgins, J. P. T., & Thompson, S. G. (2002). Quantifying heterogeneity in a meta-analysis. Statistics in Medicine, 21(11), 1539–1558. doi: 10.1002/sim.1186 12111919

[r29] Kelleher, I. (2023). Psychosis prediction 2.0: Why child and adolescent mental health services should be a key focus for schizophrenia and bipolar disorder prevention research. The British Journal of Psychiatry, 222(5), 185–187. doi: 10.1192/bjp.2022.193 36632815

[r30] Koutsouleris, N., Dwyer, D. B., Degenhardt, F., Maj, C., Urquijo-Castro, M. F., Sanfelici, R., … Meisenzahl, E. (2021). Multimodal machine learning workflows for prediction of psychosis in patients with clinical high-risk syndromes and recent-onset depression. JAMA Psychiatry, 78(2), 195. doi: 10.1001/jamapsychiatry.2020.3604 33263726 PMC7711566

[r31] LandTech. (2024). London: Population changes, density and migration patterns. London: LandTech.

[r32] Lång, U., Ramsay, H., Yates, K., Veijola, J., Gyllenberg, D., Clarke, M. C., … Kelleher, I. (2022). Potential for prediction of psychosis and bipolar disorder in Child and Adolescent Mental Health Services: A longitudinal register study of all people born in Finland in 1987. World Psychiatry, 21(3), 436–443. doi: 10.1002/wps.21009 36073707 PMC9453911

[r33] Malla, A., de Bonneville, M., Shah, J., Jordan, G., Pruessner, M., Faridi, K., … Joober, R. (2018). Outcome in patients converting to psychosis following a treated clinical high risk state. Early Intervention in Psychiatry, 12(4), 715–719. doi: 10.1111/eip.12431 28613411

[r34] Miller, J. J. (1978). The inverse of the freeman – Tukey double arcsine transformation. The American Statistician, 32(4), 138–138. doi: 10.1080/00031305.1978.10479283

[r35] Miller, T. J., McGlashan, T. H., Rosen, J. L., Cadenhead, K., Ventura, J., McFarlane, W., … Woods, S. W. (2003). Prodromal assessment with the structured interview for prodromal syndromes and the scale of prodromal symptoms: Predictive validity, interrater reliability, and training to reliability. Schizophrenia Bulletin, 29(4), 703–715. doi: 10.1093/oxfordjournals.schbul.a007040 14989408

[r36] Morgan, C., Abdul-Al, R., Lappin, J. M., Jones, P., Fearon, P., Leese, M., … Murray, R. (2006). Clinical and social determinants of duration of untreated psychosis in the ÆSOP first-episode psychosis study. British Journal of Psychiatry, 189(5), 446–452. doi: 10.1192/bjp.bp.106.021303 17077436

[r37] Mortensen, P. B., Pedersen, M. G., & Pedersen, C. B. (2010). Psychiatric family history and schizophrenia risk in Denmark: Which mental disorders are relevant? Psychological Medicine, 40(2), 201–210. doi: 10.1017/S0033291709990419 19607751

[r38] Munn, Z., Moola, S., Lisy, K., Riitano, D., & Tufanaru, C. (2015). Methodological guidance for systematic reviews of observational epidemiological studies reporting prevalence and cumulative incidence data. International Journal of Evidence-Based Healthcare, 13(3), 147–153. doi: 10.1097/XEB.0000000000000054 26317388

[r39] Newcombe, R. G. (1998). Two-sided confidence intervals for the single proportion: Comparison of seven methods. Statistics in Medicine, 17(8), 857–872. doi: 10.1002/(SICI)1097-0258(19980430)17:8<857::AID-SIM777>3.0.CO;2-E 9595616

[r40] Olin, S. -c. S., & Mednick, S. A. (1996). Risk factors of psychosis: Identifying vulnerable populations premorbidly. Schizophrenia Bulletin, 22(2), 223–240. doi: 10.1093/schbul/22.2.223 8782283

[r41] Page, M. J., Moher, D., Bossuyt, P. M., Boutron, I., Hoffmann, T. C., Mulrow, C. D., … McKenzie, J. E. (2021). PRISMA 2020 explanation and elaboration: Updated guidance and exemplars for reporting systematic reviews. BMJ, n160. doi: 10.1136/bmj.n160 33781993 PMC8005925

[r42] Papmeyer, M., Aston, J., Everts-Graber, J., Heitz, U., Studerus, E., Borgwardt, S. J., … Riecher-Rössler, A. (2018). Outcome of individuals “not at risk of psychosis” and prognostic accuracy of the Basel Screening Instrument for Psychosis (BSIP). Early Intervention in Psychiatry, 12(5), 907–914. doi: 10.1111/eip.12401 28429847

[r43] Peralta, D., Studerus, E., Andreou, C., Beck, K., Ittig, S., Leanza, L., … Riecher-Rössler, A. (2019). Exploring the predictive power of the unspecific risk category of the Basel Screening Instrument for Psychosis. Early Intervention in Psychiatry, 13(4), 969–976. doi: 10.1111/eip.12719 30019850

[r44] Pollard, T., & Howard, N. (2021). Mental healthcare for asylum-seekers and refugees residing in the United Kingdom: A scoping review of policies, barriers, and enablers. International Journal of Mental Health Systems, 15(1), 60. doi: 10.1186/s13033-021-00473-z 34127043 PMC8201739

[r45] Rasic, D., Hajek, T., Alda, M., & Uher, R. (2014). Risk of mental illness in offspring of parents with schizophrenia, bipolar disorder, and major depressive disorder: A meta-analysis of family high-risk studies. Schizophrenia Bulletin, 40(1), 28–38. doi: 10.1093/schbul/sbt114 23960245 PMC3885302

[r46] Schultze-Lutter, F., Klosterkötter, J., & Ruhrmann, S. (2014). Improving the clinical prediction of psychosis by combining ultra-high risk criteria and cognitive basic symptoms. Schizophrenia Research, 154(1–3), 100–106. doi: 10.1016/j.schres.2014.02.010 24613572

[r47] Schultze-Lutter, F., Michel, C., Schmidt, S. J., Schimmelmann, B. G., Maric, N. P., Salokangas, R. K. R., … Klosterkötter, J. (2015a). EPA guidance on the early detection of clinical high risk states of psychoses. European Psychiatry, 30(3), 405–416. doi: 10.1016/j.eurpsy.2015.01.010 25735810

[r48] Schultze-Lutter, F., Michel, C., Schmidt, S. J., Schimmelmann, B. G., Maric, N. P., Salokangas, R. K. R., … Klosterkötter, J. (2015b). EPA guidance on the early detection of clinical high risk states of psychoses. European Psychiatry, 30(3), 405–416. doi: 10.1016/j.eurpsy.2015.01.010 25735810

[r49] Schultze-Lutter, F., Schimmelmann, B. G., & Michel, C. (2021). Clinical high-risk of and conversion to psychosis in the community: A 3-year follow-up of a cohort study. Schizophrenia Research, 228, 616–618. doi: 10.1016/j.schres.2020.11.032 33234428

[r50] Schultze-Lutter, F., Walger, P., Franscini, M., Traber-Walker, N., Osman, N., Walger, H., … Michel, C. (2022). Clinical high-risk criteria of psychosis in 8–17-year-old community subjects and inpatients not suspected of developing psychosis. World Journal of Psychiatry, 12(3), 425–449. doi: 10.5498/wjp.v12.i3.425 35433326 PMC8968502

[r51] Sidik, K., & Jonkman, J. N. (2002). A simple confidence interval for meta-analysis. Statistics in Medicine, 21(21), 3153–3159. doi: 10.1002/sim.1262 12375296

[r52] Signorini, G., Singh, S. P., Boricevic-Marsanic, V., Dieleman, G., Dodig-Ćurković, K., Franic, T., … de Girolamo, G. (2017). Architecture and functioning of child and adolescent mental health services: A 28-country survey in Europe. The Lancet Psychiatry, 4(9), 715–724. doi: 10.1016/S2215-0366(17)30127-X 28596067

[r53] Steele, L., Dewa, C., & Lee, K. (2007). Socioeconomic status and self-reported barriers to mental health service use. The Canadian Journal of Psychiatry, 52(3), 201–206. doi: 10.1177/070674370705200312 17479529

[r54] Sullivan, S. A., Kounali, D., Cannon, M., David, A. S., Fletcher, P. C., Holmans, P., … Zammit, S. (2020). A population-based cohort study examining the incidence and impact of psychotic experiences from childhood to adulthood, and prediction of psychotic disorder. American Journal of Psychiatry, 177(4), 308–317. doi: 10.1176/appi.ajp.2019.19060654 31906710

[r55] Thompson, A., Marwaha, S., & Broome, M. R. (2016). At-risk mental state for psychosis: Identification and current treatment approaches. BJPsych Advances, 22(3), 186–193. doi: 10.1192/apt.bp.115.015487

[r56] Uher, R., Pavlova, B., Radua, J., Provenzani, U., Najafi, S., Fortea, L., … Fusar-Poli, P. (2023). Transdiagnostic risk of mental disorders in offspring of affected parents: A meta-analysis of family high-risk and registry studies. World Psychiatry, 22(3), 433–448. doi: 10.1002/wps.21147 37713573 PMC10503921

[r57] Veijola, J., Mäki, P., Jääskeläinen, E., Koivukangas, J., Moilanen, I., Taanila, A., … Miettunen, J. (2013). Young people at risk for psychosis: Case finding and sample characteristics of the Oulu Brain and Mind Study. Early Intervention in Psychiatry, 7(2), 146–154. doi: 10.1111/j.1751-7893.2012.00360.x 22672385

[r58] Wells, G., Shea, B., O’Connell, D., Peterson, J., Welch, V., Losos, M., & Tugwell, P. (2021). The Newcastle-Ottawa Scale (NOS) for assessing the quality of nonrandomised studies in meta-analyses. Retrieved 15 August 2024, from https://www.ohri.ca/programs/clinical_epidemiology/oxford.asp

[r59] Welsh, P., & Tiffin, P. A. (2014). The ‘At-Risk Mental State’ for psychosis in adolescents: Clinical presentation, transition and remission. Child Psychiatry & Human Development, 45(1), 90–98. doi: 10.1007/s10578-013-0380-z 23584729

[r60] World Health Organization. (1992). The ICD-10 classification of mental and behavioural disorders: Clinical descriptions and diagnostic guidelines. Geneva: World Health Organization.

[r61] Yung, A. R., & Nelson, B. (2013). The ultra-high risk concept—a review. The Canadian Journal of Psychiatry, 58(1), 5–12. doi: 10.1177/070674371305800103 23327750

[r62] Yung, A. R., Nelson, B., Stanford, C., Simmons, M. B., Cosgrave, E. M., Killackey, E., … McGorry, P. D. (2008). Validation of “prodromal” criteria to detect individuals at ultra high risk of psychosis: 2 year follow-up. Schizophrenia Research, 105(1–3), 10–17. doi: 10.1016/j.schres.2008.07.012 18765167

[r63] Yung, A. R., Stanford, C., Cosgrave, E., Killackey, E., Phillips, L., Nelson, B., & McGorry, P. D. (2006). Testing the Ultra High risk (prodromal) criteria for the prediction of psychosis in a clinical sample of young people. Schizophrenia Research, 84(1), 57–66. doi: 10.1016/j.schres.2006.03.014 16630707

[r64] Yung, A. R., Yung, A. R., Pan Yuen, H., Mcgorry, P. D., Phillips, L. J., Kelly, D., … Buckby, J. (2005). Mapping the onset of psychosis: The comprehensive assessment of at-risk mental states. Australian & New Zealand Journal of Psychiatry, 39(11–12), 964–971. doi: 10.1080/j.1440-1614.2005.01714.x 16343296

