# Supplementary material for: Sensitivity of the clinical high-risk and familial high-risk approaches for psychotic disorders – a systematic review and meta-analysis
Source: Psychol Med. 2025 Feb 12;55:e46. doi: 10.1017/S0033291724003520 (PMC12055029; doi:10.1017/S0033291724003520)
Supplement: Talukder et al. supplementary material [file S0033291724003520sup001.docx]

Supplemental sections

[Supplement 1: Search strategy for bibliographic databases 2](#_Toc179364066)

[Supplement 2: Modification made to the risk-of-bias assessment tool 3](#_Toc179364067)

[Supplement 3: Risk-of-bias assessment 4](#_Toc179364068)

[Supplement 4: Sensitivity of clinical high-risk approach based on studies involving ‘real world’ clinical high-risk services 5](#_Toc179364069)

[Supplement 5: Sensitivity of familial high-risk approach based on studies with a low risk of bias 5](#_Toc179364070)

## Supplement 1: Search strategy for bibliographic databases

#### MEDLINE (Searched via Ovid – from 1946 to September, 2024)

1. ("psychosis" OR "psychotic" OR "schizophreni*").mp.
2. exp "Schizophrenia Spectrum and Other Psychotic Disorders"/
3. 1 OR 2
4. (“at risk mental state” OR “ultrahigh risk” OR “ultra high risk” OR “clinical high risk” OR "familial high risk" OR "familial risk" OR "first degree relative*").mp
5. 3 AND 4

#### Embase (Searched via Ovid – from 1947 to September, 2024)

1. ("psychosis" or "psychotic" or "schizophreni*").mp.
2. exp psychosis/
3. exp schizophrenia spectrum disorder/
4. 1 OR 2 OR 3
5. (“at risk mental state” OR “ultrahigh risk” OR “ultra high risk” OR “clinical high risk” OR "familial high risk" OR "familial risk" OR "first degree relative*").mp
6. 4 AND 5

#### PsychINFO (Searched via Ovid – from 1806 to September, 2024)

1. (psychosis OR psychotic OR schizophreni*).mp.
2. exp Psychosis/
3. 1 OR 2
4. (“at risk mental state” OR “ultrahigh risk” OR “ultra high risk” OR “clinical high risk” OR "familial high risk" OR "familial risk" OR "first degree relative*").mp
5. 3 AND 4

#### Web of Science (Core Collection) (Searched via Clarivate – from 1900 to September, 2024)

1. ("psychosis" OR "psychotic" OR “schizophreni*”).mp
2. (“at risk mental state” OR “ultrahigh risk” OR “ultra high risk” OR “clinical high risk” OR "familial high risk" OR "familial risk" OR "first degree relative*").mp
3. 1 AND 2

## Supplement 2: Modification made to the risk-of-bias assessment tool

The Newcastle-Ottawa Quality Assessment Form for Cohort Studies (Wells et al., 2021) is designed to assess studies in three domains: 1) selection, 2) comparability, and 3) outcome. While the original tool assesses the comparability of cohorts on the basis of whether the study design or analysis controlled for specific confounders, it was not relevant to our review question to assess comparability. Therefore, we restricted the risk-of-bias assessment to the selection and outcome domains only. This changed the total possible score from 9 to 7.

We utilised the following recommended thresholds (Wells et al., 2021) for grading the studies according to the degree of risk of bias.

Low risk of bias: 3 or 4 stars in selection domain AND 2 or 3 stars in outcome domain

Moderate risk of bias: 2 stars in selection domain AND 2 or 3 stars in outcome domain

High risk of bias: 0 or 1 star in selection domain OR 0 or 1 star in outcome domain

## Supplement 3: Risk-of-bias assessment

| **Domain**  **Study** | **Selection** | | | | **Outcome** | | | **Total Score** | **Risk-of-bias grade** |
| --- | --- | --- | --- | --- | --- | --- | --- | --- | --- |
|  | **Representativeness of the exposed cohort** | **Selection of the non-exposed cohort** | **Ascertainment of exposure** | **Demonstration that outcome of interest was not present at start of study** | **Assessment of the outcome** | **Was follow-up long enough for outcomes to occur** | **Adequacy of follow up of cohorts** |  |  |
| Burke et al., 2022 | - | - | * | * | - | * | - | **3** | **High** |
| Fusar-Poli et al., 2017 | - | - | * | * | - | * | - | **3** | **High** |
| Sullivan et al., 2020 | - | * | * | * | * | * | - | **5** | **Low** |
| Blomström et al., 2016 | * | * | * | - | * | * | * | **6** | **Low** |
| Debost et al., 2019 | * | * | * | * | * | * | * | **7** | **Low** |
| Healy et al., 2024 | * | * | * | * | * | * | * | **7** | **Low** |
| Veijola et al., 2013 | - | * | * | - | * | * | - | **4** | **Moderate** |

***Note:*** *Modified Newcastle-Ottawa Quality Assessment Form for Cohort Studies; total scores represent total number of stars (*)*

## Supplement 4: Sensitivity of clinical high-risk approach based on studies involving ‘real world’ clinical high-risk services

***Note:*** *Random Effects Sidik–Jonkman Model; θ: true sensitivity parameter; CHR = Clinical high-risk.*

## Supplement 5: Sensitivity of familial high-risk approach based on studies with a low risk of bias

***Note:*** *Random Effects Sidik–Jonkman Model; θ: true sensitivity parameter; FHR = familial high-risk*
